# Extrapolating Prognostic Factors of Primary Curative Resection to Postresection Recurrences Hepatocellular Carcinoma Treatable by Radiofrequency Ablation

**DOI:** 10.1155/2021/8878417

**Published:** 2021-01-02

**Authors:** Hui Ma, Zhongchen Li, Jia Yuan, Lan Zhang, Xiaoying Xie, Xin Yin, Rongxin Chen, Zhenggang Ren

**Affiliations:** Liver Cancer Institute, Zhongshan Hospital, Fudan University, 180 Fenglin Road, Shanghai 200032, China

## Abstract

**Objective:**

Recurrence after curative resection for hepatocellular carcinoma (HCC) is a major cause of death from this disease. Factors of primary curative resection are available and potential in the prognosis of follow-up treatment. Our aim was to assess the prognostic significance of primary curative resection factors in recurrent HCC patients undergoing radiofrequency ablation therapy (RFA).

**Methods:**

In this retrospective study, we assessed 235 patients who underwent limited RFA of HCC recurrences (tumors ≤ 5 cm; nodules ≤ 3) after primary curative resection. Factors of primary curative resection were collected, and overall survival and recurrence-free survival were evaluated by the Kaplan-Meier method. Univariate and multivariate analyses were used to identify significant prognostic factors.

**Results:**

After a median follow-up of 36 months, 54 patients died, and 128 patients had hepatic recurrence. On univariate analyses, patients whose primary tumors were less differentiated (*p* = 0.032 and *p* = 0.048) and required less time to recur (*p* = 0.013 and *p* = 0.001) after curative resection displayed poorer overall survival and higher recurrence rates following RFA. On multivariate analyses, the pathologic tumor grade (*p* = 0.026 and *p* = 0.038) and recurrence-free survival after primary curative resection (*p* = 0.028 and *p* < 0.001) emerged as independent risk factors of survival and HCC recurrence.

**Conclusions:**

Primary tumor differentiation and time to recurrence after curative resection are viable prognostic factors of overall survival and further recurrence risk in patients undergoing RFA of recurrent HCC.

## 1. Introduction

Hepatocellular carcinoma is the fourth most common cause of cancer-related death worldwide [1]. Surgical resection represents one of the best first-line treatments for selected patients [2]. However, recurrences after curative surgery are frequent (range, 40-70%) [3, 4]. Proper follow-up for postoperative patients and recent advances in diagnostic modalities has led to an increased detection rate of recurrent tumors at an early stage. Specifically, these tumors are solitary and small, and they represent an opportunity for radical treatment.

Compared with initial treatment, more limited liver function reserve and technical difficulties in repeated hepatic resection owing to postoperative adhesion are expected in the treatment of recurrent HCC after hepatic resection [5]. Radiofrequency ablation (RFA) is regarded as an alternative curative treatment modality for small recurrent HCC instead of repeated surgical resection because of minimal invasion and damage to the liver, and some studies showed that RFA had similar outcomes as repeated surgical resection [5–7]. However, even complete tumor ablation does not ensure disease eradication. It is estimated that the cumulative 5-year recurrence rate of patients undergoing RFA is more than 70% [5–7]. To improve the long-term outcome of RFA, it is crucial to elucidate the mechanisms and risk factors associated with prognosis after RFA.

Factors of primary curative resection are available for recurrent HCC patients without additional traumatic examination, including detailed clinical and pathological information of primary tumors, which have been shown as prognostic factors related to overall survival and recurrence of primary curative resection [8–10]. However, their clinical merits in recurrent HCC patients undergoing RFA have yet to be proven.

In the present study, we try to explore the significance of factors of primary curative resection in prognosis in recurrent HCC after treatment with RFA. Therefore, we performed a study on recurrent HCC cases after curative resection treated with RFA and investigated the relationship between factors of primary curative resection and prognosis including both overall survival and tumor recurrence after treatment.

## 2. Patients and Methods

### 2.1. Ethical Approval

All procedures involving human participants maintained standards of the Ethics Committee of Zhongshan Hospital affiliated with Fudan University, the 1964 Helsinki Declaration and its later amendments, or comparable ethical principles. The need for formal informed consent of individual participants was waived, because no patients were at risk in the retrospective analysis. Patient records were anonymized and deidentified prior to analysis.

### 2.2. Patient Selection

We conducted a retrospective analysis of prospectively collected data, contributed by 623 consecutive patients with recurrences of HCC after primary curative resection. All had undergone RFA at the Liver Cancer Institute of Zhongshan Hospital (Fudan University) between January 2010 and December 2015. In each instance, for primary tumors, the diagnosis was based on histologic assessment; for recurrent HCC, diagnosis was based on the histologic result or criteria established by the American Association for the Study of Liver Disease (AASLD). Typically, HCC is marked by intense arterial uptake of contrast agent and subsequent washout in venous-delayed phases of contrast-enhanced magnetic resonance imaging (MRI), or computed tomography (CT) [11].

Only 305 of the initial 623 study candidates met our inclusion criteria as follows: (1) no prior treatment of HCC other than curative resection; (2) time to recurrence ≥ 3 months after curative resection; (3) Child-Pugh class A or B liver function; (4) limited recurrent disease (single nodule ≤ 5.0 cm or ≤3 nodules, the largest ≤ 3.0 cm); and (5) no invasion of major intrahepatic vessels or extrahepatic metastasis. Another 70 patients were excluded due to incomplete ablation (*n* = 5) or loss of follow-up (*n* = 65) within 12 months. Incomplete ablation was defined as CT or MRI evidence of irregular, peripherally enhancing foci in ablation zones 4 weeks after RFA, in the absence of salvage RFA. Ultimately, 235 patients were selected for study.

### 2.3. RFA Procedures

Patients were treated either with the RITA RFA system (Starburst XL; Mountain View, CA, USA) or Cool-Tip RFA system (Covidien; Boulder, CO, US) to deliver the RF energy [12]. The procedures were performed percutaneously with real-time ultrasonic guidance, with patients pretreated with local anesthetic and intramuscular sedation. On withdrawal of the electrode needle, the tract was ablated to prevent bleeding and tumor seeding. Cardiovascular and respiratory functions were monitored, and the hyperechoic area around the electrode tip was observed by ultrasonic monitoring during the procedure. The treatment was designed to cover the whole tumor area with at least a 5 mm safety ablative margin extending into the surrounding normal hepatic parenchyma. One month after RFA, contrast-enhanced MRI or CT was performed to evaluate the effectiveness of tumor ablation. Patients with residual tumor were treated by salvage RFA.

### 2.4. Follow-Up Observation after RFA Treatment

The median follow-up period was 36 months (range, 11-82 months). Patients were followed up with an interval of 2-3 months. Serum alpha-fetoprotein (AFP) levels were measured, liver function was analyzed, and ultrasonography and CT or MRI was performed. If recurrent HCC was again detected (confirmed by typical imaging features), patients were managed accordingly, opting for repeated RFA, surgical resection, percutaneous ethanol injection, transarterial chemoembolization (TACE), or radiotherapy. Procedure-related mortality was defined as any death occurring within 30 days after RFA. Recurrence-free survival (RFS) was calculated from the date of RFA until the time at which tumor recurrence was confirmed. Patients were censored at date of death or at date of last follow-up visit if tumor recurrence was not diagnosed.

### 2.5. Factors Prognostic of Primary Curative Resection Outcomes

Factors of primary curative resection were collected including primary tumor size, tumor number, tumor differentiation, microvascular invasion or not, encapsulation invasion or not, and star lesion or not, according to the postoperative pathological report. And postoperative adjuvant TACE and time to relapse after curative resection were also included on the basis of follow-up data.

### 2.6. Statistical Analyses

Analysis was performed with SPSS 19.0 for Windows (SPSS; Chicago, IL, USA). All consecutive data were expressed as mean ± standard deviation. Differences between the two groups were analyzed using the unpaired *t*-test for continuous variables, and categorical variables were analyzed using the *x*^2^ test or Fisher's exact test. The cumulative overall survival (OS) rate and RFS rate were assessed by the Kaplan-Meier method, and the difference between the two groups was evaluated with the log-rank method. Prognostic factor affecting OS and RFS was determined using univariate and multivariate analysis: significant variables obtained by univariate analysis were tested by multivariate analysis using Cox's proportional hazard model. A *p* value of <0.05 was considered to be statistically significant.

## 3. Results

The median age of patients under study was 56 years (range, 21-83 years). There were 196 men and 39 women (male-to-female ratio, 5.0 : 1). Most patients (90.21%, 212/235) tested positive for hepatitis B virus (HBV) infection, rarely (0.85%, 2/235) testing positive for hepatitis C virus (HCV) infection, and none showed dual HBV/HCV positivity. Based on the Child-Pugh classification of the liver function, 95.74% (225/235) HCC patients were at class A liver function and 4.26% (10/235) at class B. After a median follow-up of 36 months, 128 patients had developed recurrences, and 55 deaths were recorded. OS rates at 1, 3, and 5 years were 99.1%, 78.2%, and 61.2%, respectively. Corresponding RFS rates were 60.9%, 44.8%, and 35.8%, respectively.

Analysis of prognostic factors affecting OS and RFS was performed. The variables tested by univariate analysis were sex, age, hepatitis history, factors of primary curative resection, and features before RFA including total bilirubin (TB), alanine aminotransferase (ALT), prothrombin time (PT), albumin, *γ*-glutamyltranspeptidase (GGT), AFP, tumor size, and tumor number. The results indicated that primary tumor differentiation, time to recurrence after curative resection, GGT, and AFP before RFA were significant prognostic factors associated with OS; while primary tumor differentiation, time to recurrence after curative resection, GGT before RFA, and recurrent tumor number after curative resection associated with RFS (Table 1). All significant parameters and related variables were then subjected to multivariate Cox proportional hazards analysis. Primary tumor differentiation, time to recurrence after curative resection, GGT, and AFP before RFA were found to be independent risk factors of OS (Table 2), and primary tumor differentiation, time to recurrence after curative resection, GGT before RFA, and recurrent tumor number after curative resection were independent risk factors of RFS (Table 3).

**Figure 1:**
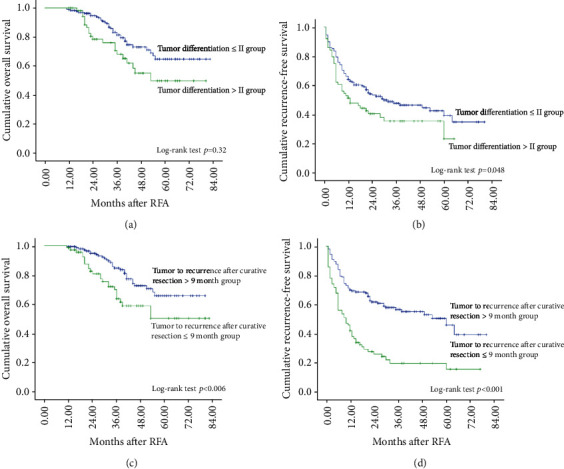
Worse primary tumor differentiation and short time to recurrence after curative resection bode poorly for clinical outcomes of recurrent hepatocellular carcinoma undergoing radiofrequency ablation. Univariate analyses of primary tumor differentiation demonstrated that worse tumor differentiation was associated with poorer overall survival (OS) rates (a) and poorer recurrence-free survival (RFS) rates (b) (Kaplan-Meier and log-rank tests). Prognostic performance of primary tumor differentiation was significant for both OS and RFS (*p* = 0.032 and *p* = 0.048, respectively). Univariate analyses of time to recurrence after curative resection demonstrated that short time to recurrence was associated with poorer OS rates (c) and poorer RFS rates (d) (Kaplan-Meier and log-rank tests). Prognostic performance of time to recurrence after curative resection was significant for both OS and RFS (*p* = 0.006 and *p* < 0.001, respectively).

Primary tumor differentiation was proven significant by univariate analysis in predicting both OS and RFS, using ≤ grade II vs > grade II tumor differentiation for dichotomous evaluation (*p* = 0.032, *p* = 0.048, respectively) (Figures 1(a) and 1(b)). In multivariate models, primary tumor differentiation emerged as independently predictive of OS (hazard ratio [HR] = 1.878, 95% confidence interval [CI]: 1.077-3.275; *p* = 0.026) and recurrence (HR = 1.497, 95% CI: 1.022–2.192; *p* = 0.038).

Time to recurrence after curative resection was proven significant by univariate analysis in predicting both OS and RFS, using >9 months vs ≤9 months to recurrence after curative resection for dichotomous evaluation (*p* = 0.013 and *p* = 0.001, respectively) (Figures 1(c) and 1(d)). In multivariate models, time to recurrence after curative resection emerged as independently predictive of OS (HR = 1.828, 95% CI: 1.066-3.135; *p* = 0.028) and recurrence (HR = 2.400, 95% CI: 1.687-3.414; *p* < 0.001). Furthermore, *p* values were calculated for log-rank survival analysis using different cut-offs for time to recurrence after curative resection. *p* values reflected statistical significance when using 9 and 12 months as cut-off values in OS analysis and 4 to 36 months in recurrence-free survival analysis, suggesting good reproducibility (Table 4).

Baseline clinical characteristics of patients experiencing short and long times to recurrences after curative resection are listed in Table 5, and the distribution are shown in Figure S1. Shorter time to recurrence after curative resection was closely linked with less differentiated primary tumor and primary tumor microvascular invasion.

## 4. Discussion

RFA is a safe and effective treatment for HCC patients with solitary and small recurrent tumors of the liver after curative resection, especially for those who are not candidates for repeat resection [4, 5]. Unfortunately, recurrent HCC patients undergoing RFA, which is designed to destroy tumors locally without damage to the surrounding tissue, also have a high incidence of recurrence after completing percutaneous ablation [5–7]. Typically in HCC, most deaths are due to tumor recurrence or local progression [13]. Although factors of primary curative resection, including detailed clinical and pathological information of primary tumors, have been shown as prognostic factors related to overall survival and recurrence of primary curative resection [8–10], few studies have focused on the relationship between these factors and prognosis for recurrent HCC patients undergoing RFA. The prognostic significance of factors of primary curative resection, which are available for recurrent HCC patients without additional traumatic examination, would be very attractive for use in the clinic. In the present study, we found that worse curative resection tumor differentiation and short time to recurrence after curative resection were independently related to poorer survival and higher another recurrence incidence of patients with recurrent HCC undergoing RFA.

Worse tumor differentiation has been shown to be a poor prognostic factor related to the survival and recurrent rate in HCC patients undergoing curative resection [8–10]. In this study, our results showed that worse tumor differentiation is still predictive of poorer survival and increased risk of further recurrences, even in patients undergoing RFA of recurrent HCC. As RFA destroys tumors locally without removing tumors, pathology results of recurrent HCC undergoing RFA usually are not available. Therefore, whether the differentiation of recurrent HCC tumors is related to primary tumors has not been analyzed and needs to be further studied.

Time to recurrence after curative resection was an independent prognostic factor for outcome after recurrent tumors undergoing RFA in this study, which was similar to a previous report in recurrent HCC patients after curative resection undergoing second resection [14]. Time to recurrence was related to the origin of intrahepatic recurrence, either intrahepatic metastasis or multicentric occurrence [15, 16]. A study by Kumada in fact has shown that the incidence of intrahepatic metastasis in the 1st, 2nd, 3rd, 4th, and 5th years after resection or percutaneous ethanol injection treatment for HCC was 17.8%, 17.1%, 6.9%, 0%, and 4.4%, respectively; the incidence of multicentric occurrence in the 1st, 2nd, 3rd, 4th, and 5th year was 5.3%, 14.4%, 9.2%, 8.6%, and 13.3%, respectively, which supported the idea that intrahepatic recurrence within one postoperative year may mainly originate from intrahepatic metastasis, while it was from multicentric occurrence after three postoperative years [15]. In this study, when using 9 months or 12 months as the cut-off value of time to recurrence after curative resection, *p* value was statistically significant in both overall survival and recurrence free survival analysis, and results suggested that short time to recurrence after curative resection was related to worse tumor differentiation and presence of microvascular invasion, indicating that poorer survival of patients with recurrent HCC undergoing RFA was mainly related to intrahepatic metastasis. Consequently, a strategy incorporating other measures to prevent such metastasis may potentially improve the survival rates of patients undergoing RFA of recurrent HCC.

In addition to factors extrapolated from primary curative resection, baseline serum GGT concentration (i.e., prior to RFA) was identified to be an independent risk factor of OS and RFS for recurrent HCC patients undergoing RFA, consistent with our previous results that the GGT level was a serum marker that may be used for prognosis in HCC treated by RFA [12]. The number of recurrent hepatic nodules present was also recognized as an independent risk factor for RFS in patients undergoing RFA of recurrent HCC. However, the tumor size was not prognostic of OS or RFS, given perhaps the limited number of patients with large tumors in this study.

In summary, the data analyzed herein indicate that primary tumor differentiation and time to recurrence after curative resection are predictive of OS and repeat postoperative recurrences of HCC in patients destined for RFA. These factors can be used as a routine assessment of such HCC patients in order to support intensive follow-up observations and to optimize management.

## Figures and Tables

**Table 1:** Univariate analysis of the prognostic factors contributing to OS and RFS of recurrent HCC patients undergoing RFA.

	Variables	n	OS *p* value^1^	RFS *p* value^1^
Factors of primary curative resection	Gender, male/female	196/39	0.443	0.111
	Age (>55years), yes/no	120/115	0.581	0.769
	Hepatitis, yes/no	214/21	0.306	0.321

Factors of primary curative resection	Tumor size (>3 cm), yes/no	127/108	0.652	0.592
	Tumor number, multiple/single	51/184	0.729	0.319
	Tumor differentiation (>II), yes/no	64/171	0.032	0.048
	Microvascular invasion, yes/no	56/179	0.380	0.289
	Encapsulation invasion, yes/no	7/228	0.672	0.216
	Star lesion, yes/no	6/229	0.650	0.051
	Postoperative adjuvant TACE, yes/no	69/166	0.050	0.101
	Time to recurrence after curative resection (≤9 months), yes/no	78/157	0.006	<0.001

Features before RFA	TB (>17.1 *μ*mol/l), yes/no	30/205	0.129	0.084
	ALT (>50 IU/l), yes/no	32/203	0.375	0.718
	PT (>14 s), yes/no	14/221	0.066	0.979
	Albumin (≤3.5 g/dl), yes/no	12/223	0.781	0.361
	GGT (>50 IU/l), yes/no	95/140	0.002	0.022
	AFP (>20 ng/ml), yes/no	80/155	0.010	0.292
	Tumor size (>2.0 cm), yes/no	72/163	0.830	0.853
	Tumor number, multiple/single	36/199	0.622	0.023

OS: overall survival; RFS: recurrence-free survival; RFA: radiofrequency ablation; TB: total bilirubin; ALT: alanine aminotransferase; PT: prothrombin time; GGT: *γ*-glutamyltranspeptidase; AFP: alpha-fetoprotein; ^1^log-rank test.

**Table 2:** Multivariate analysis of the factors associated with OS of recurrent HCC patients undergoing RFA.

Risk factor	Hazard ratio	95% CI	*p* value^1^
Tumor differentiation (>II), yes/no	1.878	1.077-3.275	0.026
Time to recurrence after curative resection (≤9 months), yes/no	1.828	1.066-3.135	0.028
GGT (>50 IU/l), yes/no	2.186	1.271-3.760	0.005
AFP (>20 ng/ml), yes/no	1.929	1.133-3.284	0.016

OS: overall survival; RFA: radiofrequency ablation; CI: confidence interval; GGT: *γ*-glutamyltranspeptidase; AFP: alpha-fetoprotein; ^1^Cox proportional hazard model.

**Table 3:** Multivariate analysis of the factors associated with RFS of recurrent HCC patients undergoing RFA.

Risk factor	Hazard ratio	95% CI	*p* value^1^
Tumor differentiation (>II), yes/no	1.497	1.022-2.192	0.038
Time to recurrence after curative resection (≤9 months), yes/no	2.400	1.687-3.414	<0.001
GGT (>50 IU/l), yes/no	1.496	1.050-2.133	0.026
Tumor number, multiple/single	1.681	1.075-2.628	0.023

RFS: recurrence-free survival; RFA: radiofrequency ablation; CI: confidence interval; GGT: *γ*-glutamyltranspeptidase; ^1^Cox proportional hazard model.

**Table 4:** Time to recurrence after curative resection predictive value of OS and RFS using different cut-off values.

Cut-off values of time to recurrence after curative resection (months)	Log-rank analyses for OS, *p* value^1^	Log-rank analyses for RFS, *p* value^1^
4	0.636	0.001
6	0.380	<0.001
9	0.006	<0.001
12	0.013	0.001
18	0.083	<0.001
24	0.089	0.002
36	0.683	0.004

OS: overall survival; RFS: recurrence-free survival; ^1^log-rank test.

**Table 5:** Baseline characteristics between the short time to recurrence after curative resection group and the long time to recurrence after curative resection group.

	Variables	Short time to recurrence after curative resection (*n* = 78)	Long time to recurrence after curative resection (*n* = 157)	*p* value
Factors of primary curative resection	Gender, male/female	69/9	127/30	0.098^2^
	Age (years)	53.359 ± 12.732	55.950 ± 11.601	0.120^1^
	Hepatitis, yes/no	72/6	142/15	0.419^2^
	Cirrhosis, yes/no	17/61	37/120	0.449^1^
	Tumor size (cm)	4.172 ± 2.396	4.115 ± 2.687	0.874^1^
	Tumor number, multiple/single	17/61	34/123	0.553^2^
	Tumor differentiation (>II), yes/no	28/50	36/121	0.027^2^
	Microvascular invasion, yes/no	25/53	32/125	0.029^2^
	Encapsulation invasion, yes/no	2/76	5/152	0.574^2^
	Star lesion, yes/no	2/76	4/153	0.649^2^
	Postoperative adjuvant TACE, yes/no	26/52	43/114	0.214^2^

Features before RFA	TB (>17.1 *μ*mol/l), yes/no	3/75	27/130	0.452^2^
	ALT (>50 IU/l), yes/no	12/66	20/137	0.578^2^
	PT (>14 s), yes/no	5/73	9/148	0.836^2^
	Albumin (≤3.5 g/dl), yes/no	2/76	10/147	0.212^2^
	GGT (>50 IU/l), yes/no	36/42	59/98	0.207^2^
	AFP (>20 ng/ml), yes/no	26/52	54/103	0.872^2^
	Tumor size (cm)	1.754 ± 0.613	1.698 ± 0.525	0.470^1^
	Tumor number, multiple/single	14/64	22/135	0.272^2^

Data are expressed as the number or mean value ± standard deviation. RFA: radiofrequency ablation; TB: total bilirubin; ALT: alanine aminotransferase; PT: prothrombin time; GGT: *γ*-glutamyltranspeptidase; AFP: alpha-fetoprotein; ^1^unpaired t test; ^2^Fisher's exact test or *x*^2^ test.

## Data Availability

All data are available within this manuscript or from the corresponding author upon reasonable request.

## Supplementary Materials

Supplementary Materials Figure S1: baseline characteristics (continuous variables) between the short time to recurrence (STR) after the curative resection group and the long time to recurrence (LTR) after the curative resection group.
