# Supplementary material for: Extrapolating Prognostic Factors of Primary Curative Resection to Postresection Recurrences Hepatocellular Carcinoma Treatable by Radiofrequency Ablation
Source: Gastroenterol Res Pract. 2021 Jan 2;2021:8878417. doi: 10.1155/2021/8878417 (PMC7801068; doi:10.1155/2021/8878417)
Supplement: Supplementary Materials — Figure S1: baseline characteristics (continuous variables) between the short time to recurrence (STR) after the curative resection group and the long time to recurrence (LTR) after the curative resection group. [file 8878417.f1.docx]

**Figure S1. Baseline characteristics (continuous variables) between the short time to recurrence (STR) after curative resection group and the long time to recurrence (LTR) after curative resection group.**

**
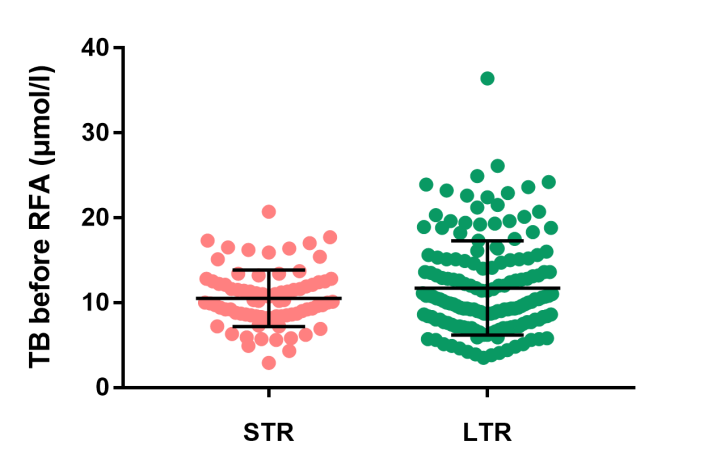

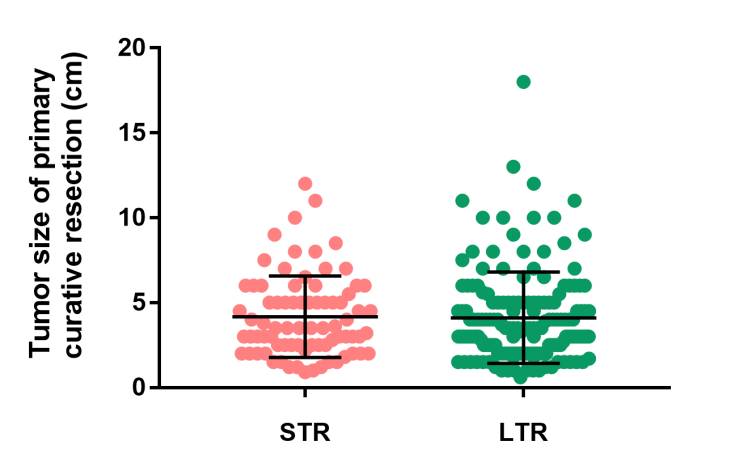

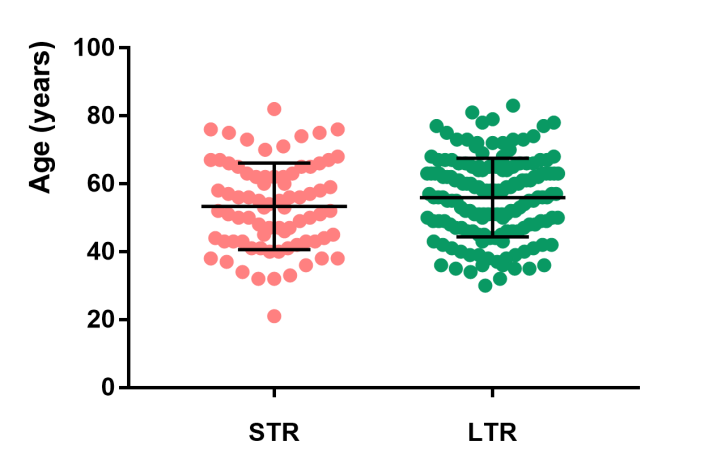
**
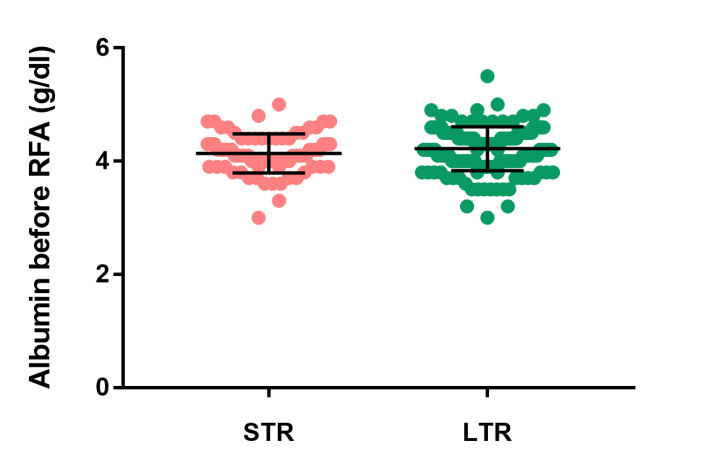

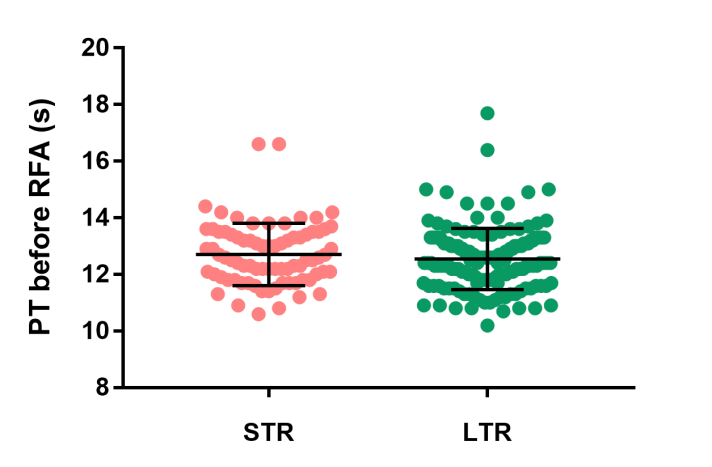

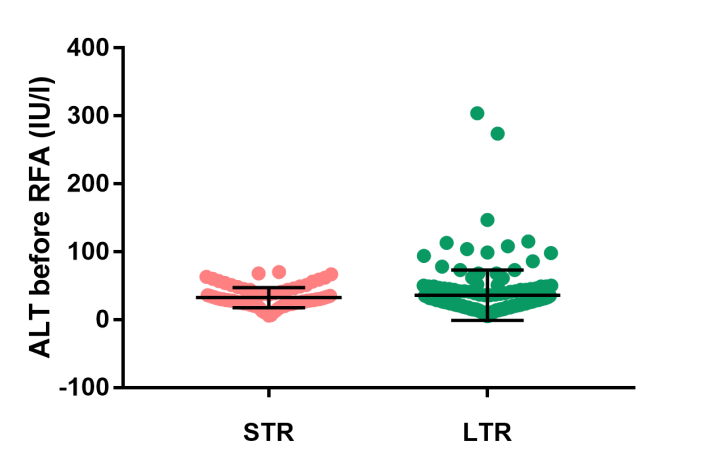


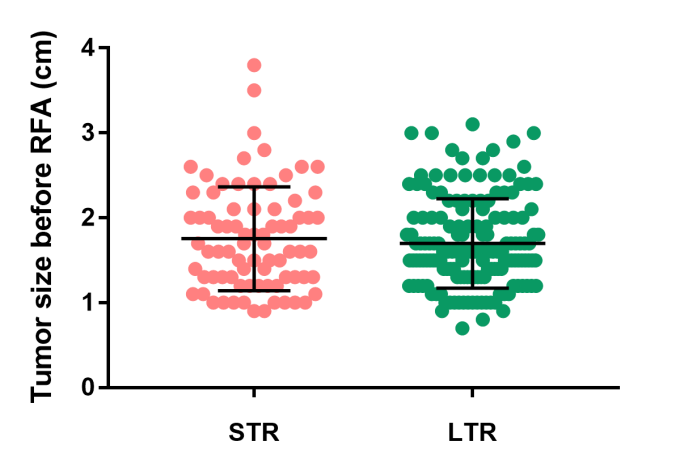

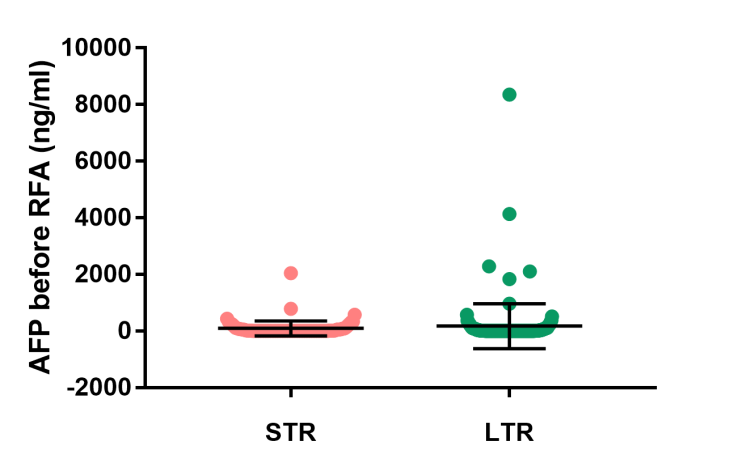

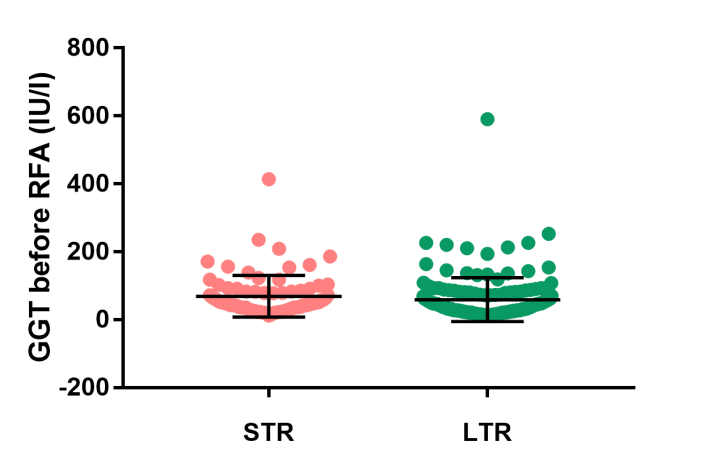


RFA, radiofrequency ablation; TB, total bilirubin; ALT, alanine aminotransferase; PT, prothrombin time; GGT, γ-glutamyltranspeptidase; AFP, alpha-fetoprotein
